# Development of Porous Titania Structure with Improved Photocatalytic Activity: Response Surface Modeling and Multi-Objective Optimization

**DOI:** 10.3390/nano10050998

**Published:** 2020-05-23

**Authors:** Elvira Mahu, Maria Ignat, Corneliu Cojocaru, Petrisor Samoila, Cristina Coromelci, Iuliean Asaftei, Valeria Harabagiu

**Affiliations:** 1 Laboratory of Materials Chemistry, Department of Chemistry, “Alexandru Ioan Cuza” University of Iasi, 11 Carol I Boulevard, 700506 Iasi, Romania; mahu.elvira@icmpp.ro (E.M.); samoila.petrisor@icmpp.ro (P.S.); cristina.pastravanu.coromelci@gmail.com (C.C.); ivasaftei@yahoo.com (I.A.); 2 Laboratory of Inorganic Polymers, “Petru Poni” Institute of Macromolecular Chemistry, 41 A Grigore Ghica Vodă Aley, 700487 Iasi, Romania; hvaleria@icmpp.ro; 3 Science Research Department, Institute for Interdisciplinary Research, “Alexandru Ioan Cuza” University of Iasi, Lascar Catargi Str. 54, 700107 Iasi, Romania

**Keywords:** porous titania, response surface methodology, optimization, photocatalytic activity

## Abstract

Porous titania was successfully synthesized by an ultrasound-assisted sol-gel route. The synthesis process was empirically modeled and optimized using the response surface methodology (RSM). Input variables adopted for optimization dealt with the weight ratio of precursors (*r*) and the sonication time (*t*), representing the used factors in the synthesis procedure. With regard to application, the synthesized TiO_2_ samples were tested for the photodegradation of two water-soluble organic pollutants under UV–Vis irradiation. Optimal conditions for the efficient pollutants’ photodegradation were found to involve a precursors ratio of 3 and a sonication time of 60 min. Thus, the M5 sample prepared under the founded optimal conditions yielded the maximal removal efficiencies of 98.4% and 46.3% for the photodegradation of CR dye and 2,4-D herbicide, respectively. In addition, the photodegradation kinetics revealed the pseudo first-order rate constants, showing the photodegradation of CR (*k_1_* = 8.86 × 10^−2^ min^−1^) by M5 sample is about 1.3-fold faster than the photodegradation of 2,4-D pesticide (*k_2_* = 6.84 × 10^−2^ min^−1^).

## 1. Introduction

In the last few decades, the problem of environmental pollution has increased in amplitude, owing to perpetual industrial progress. The presence of organic micropollutants (pesticides, pharmaceuticals, dyes, etc.) in water resources represents a serious contamination, because they are persistent, having a negative impact on the aquatic ecosystem and consequently, on human health [1].

In order to remove the water-soluble organic pollutants, various separation processes can be employed. In this regard, the conventional methods for the remediation of waters, loaded with organic contaminants, deal with coagulation–flocculation [2,3] and adsorption [4,5] processes. Despite these common techniques, research interest also has been devoted towards exploring promising technologies able to destroy the hazardous organic compounds, rather than transferring the pollutants from one phase to another [6]. Such methods are known as advanced oxidation processes (AOPs) that imply the destructive oxidation of organic pollutants [6,7,8]. Hence, AOPs deal with homogenous catalysis and photocatalysis (Fenton, H_2_O_2_/UV, Fenton-like [7,8]), heterogeneous catalysis (CWPO–Catalytic Wet Peroxide Oxidation) [9], heterogeneous photocatalysis (TiO_2_/UV) [6,8] and others (e.g., catalytic ozonation [10]). According to the AOPs mechanism, the toxic pollutants are transformed into less hazardous compounds with a diminished impact on the environment. In this context, many researchers have focused their efforts on improving several aspects of the photocatalytic processes [11]. For instance, the coupling of the photocatalytic and adsorption process can ameliorate water reclamation and reduce the operational costs [12]. Generally, oxide nanomaterials, such as titanium oxide, zinc oxide [13], copper oxide [14], and graphene oxide [15], have demonstrated their promising potential for photocatalytic degradation of the organic pollutants (phenol, bisphenol, methylene blue, 4-nitrophenol etc.). Among them, one of the most applied materials with distinguished photocatalytic properties is titanium dioxide (TiO_2_), because the significant advantages of this material are related to its non-toxicity, long-term stability, simple preparation route, adjustable structure and tailored morphology. Until now, mesoporous titania (TiO_2_) has been used in a variety of technological applications, like water purification and air cleaning systems, sterilization, self-cleaning surfaces, photoelectrochemical conversion, and catalytic hydrogen evolution [16,17].

In this study, the synthesis of porous TiO_2_ by the ultrasound-assisted sol-gel method is reported. Moreover, the multi-objective optimization of the ultrasound-assisted process to enhance the photocatalytic activity of the produced TiO_2_ porous material is focused. In this regard, the design of experiments (DoE) and response surface methodology (RSM) were employed to find out the optimal conditions for the synthesis of porous titania exhibiting improved photocatalytic activity. Thus, the influence of two factors in the synthesis procedure, as the weight ratio between precursors (*r*) and the sonication time (*t*) was evaluated. The produced material was characterized by nitrogen-sorption measurements, scanning electron microscopy (SEM), transmission electron microscopy (TEM), X-ray diffraction (XRD), Fourier-transform infrared spectroscopy (FTIR), and UV–Vis Diffuse Reflectance. The photocatalytic performances of the produced TiO_2_ were evaluated in the photodegradation processes of Congo red dye and 2,4-D herbicide, these being selected as representative organic-persistent water pollutants. Furthermore, the kinetic parameters of the photodegradation reactions of the considered pollutants were assessed.

## 2. Materials and Methods

### 2.1. Materials

Pluronic^®^ F-127 (tri-block copolymer of poly(ethylene oxide) poly(propylene oxide)-poly(ethylene oxide) (PEO_101_-PPO_56_-PEO_101_)), titanium (IV) isopropoxide (C_12_H_28_O_4_Ti, purity ≈ 97%), Congo red dye (C_32_H_22_N_6_Na_2_O_6_S_2_), and 2,4-diclhorophenoxi acetic acid (Cl_2_C_6_H_3_OCH_2_CO_2_H) were purchased from Sigma-Aldrich Chemie GmbH, Taufkirchen Germany. Isopropyl alcohol (C_3_H_8_O, ≥99.7%) was obtained from S.C. Chemical Company S.A., Iasi, România. All products were analytical grade and used as received.

### 2.2. TiO_2_ Synthesis

**Scheme 1:**
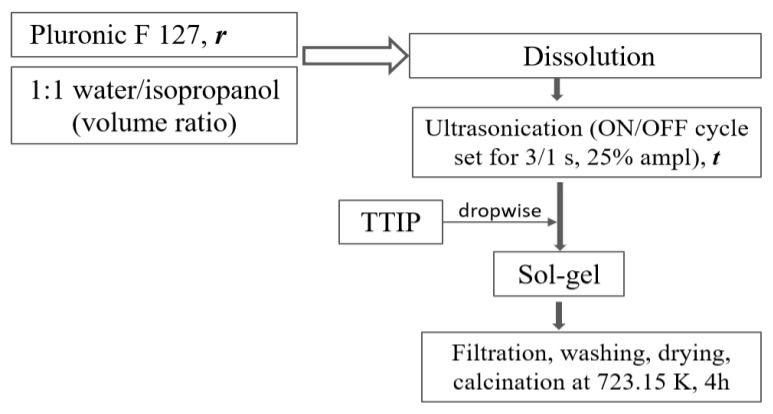
Synthesis scheme followed in the preparation procedure of TiO_2_.

Mesoporous TiO_2_ was prepared applying an ultrasound-assisted sol-gel method using titanium tetraisopropoxide (TTIP) as the titania precursor, and Pluronic^®^ F-127 as structure directing agent. Briefly, Pluronic^®^ F-127 (*r* grams, according to Table 1) was dissolved by continuous magnetic stirring in a water/isopropanol (1:1 volume ratio) mixture and subjected to the ultrasonication process for different time intervals (*t*, min), using a horn-probe sonic tip (VibraCell, 750 W) at 25% of amplitude with a pulsed ON/OFF cycle set for 3/1 s (see Scheme 1). In the first 10 min of the ultrasonication process, the well dosed quantities of TTIP were added dropwise to the alcoholic mixture of F-127, and left to hydrolyze for the remained time set. Afterward, the resulted sol was filtered, and the obtained solid was washed several times with distilled water and dried at room temperature. Further to this, F-127 molecules have been removed from the solid titania matrix by a thermal treatment, leaving behind pores through the titania structure. Thus, the dried yellow powdered samples were calcined at 723.15 K for 4 h, using a heating oven. For the optimization of the synthesis process, a series of nine samples of TiO_2_ were prepared according to the statistical technique—design of experiments (DoE)—by varying simultaneously the surfactant/titanium source weight ratios and ultrasonication time (according to Table 2).

### 2.3. Materials Characterization

Textural properties of synthesized titania samples were highlighted by nitrogen adsorption–desorption on the NOVA 2200e (Quantachrome instruments, Boynton Beach, Florida, USA) automated gas adsorption system using liquid nitrogen adsorbate (at 77.15 K). Before analysis, each sample has been outgassed for 4 h at 473.15 K, leaving behind completely empty pores. During the analysis, the adsorption–desorption isotherms have been registered, to which the BET (Brunauer–Emmett–Teller) and BJH (Barrett–Joyner–Halenda) equations have been applied in order to estimate the BET specific surface area and BJH pore size distribution. The total pore volume has been evaluated considering the relative pressure on the adsorption branch of isotherm at *P*/*P*_0_ = 0.95.

The structural characterization of the synthesized TiO_2_ nanoparticles was carried out on a Shimadzu LabX XRD-6000 (Kyoto, Japan) advanced diffractometer, in the range of 20°–80° (2 theta), using the Cu-Kα radiation, *λ* = 1.5406 Å, working in standard mode.

The morphology of titania powders was investigated using an environmental scanning electron microscope (SEM) type Quanta 200 apparatus, equipped with SE (secondary electron), and BSE (backscatter electron) detectors, working at an accelerating voltage between 20 kV, and the SEM micrographs were recorded in a low vacuum mode. Further to this, titania nanoparticles were highlighted by transmission electron microscopy (TEM) using a Hitachi HT7700 (Tokyo, Japan) microscope equipped with a Bruker XFlash 6 EDS (Manning Road Billerica, MA, USA) detector operating at 120 kV in high contrast mode. TEM images allowed the selection of an area in the polycrystalline materials in order to acquire ring patterns analogous to those from X-ray powder diffraction, which were used to identify texture and discriminate nanocrystalline from amorphous phases in titania samples.

Fourier-transform infrared spectroscopy has been used to investigate surface chemistry and FTIR spectra have been recorded on a Bruker Vertex FTIR (Manning Road Billerica, MA, USA) spectrometer, resolution of 2 cm^−1^, in the range of 4000–400 cm^−1^ by KBr (Sigma-Aldrich Chemie GmbH, Taufkirchen Germany) pellet technique.

The UV–Vis DR (UV–Vis Diffuse Reflectance) spectra were obtained with a powder UV–Vis spectrophotometer (Shimazu, UV-2450). The remission function of Kubelka–Munk (*F*(*R*) = (1 − *R*)^2^/2*R* = *k*/*s* = *Ac*/*s*, where *R* is the reflectance, *k—*absorption coefficient, *s—*scattering coefficient, *c—*concentration of the absorbing species, *A—*absorbance) was used to analyze the electronic properties of TiO_2_ samples. Afterward, the Tauc plots were drawn, from which the band gap energy (*E_g_*) was evaluated.

### 2.4. Photocatalytic Experiment

The photocatalytic activity of the produced materials was estimated by testing the degradation of CR dye and 2,4-D pesticide under UV-light irradiation using a photoreactor with a volumetric capacity of *V_R_* = 50 mL. To this end, the photodegradation tests were performed in a cylindrical photoreactor containing 50 mL of CR or 2,4-D solution (50 mg/L), to which 0.05 g of photocatalyst was added, and equipped with a magnetic stirrer and an external UV-lamp (model VL-6.LC, wavelengths of 365 nm/254 nm, 6 W). The UV-lamp was placed on top of the photoreactor vessel, above the suspension surface at a distance of 10 cm. The experiment started with reaching the adsorption–desorption equilibrium by magnetically stirring the suspension in the dark for 30 min. Afterwards, the UV-lamp was switched on and the solution was exposed up to 120 min to UV-light irradiation. The concentration of pollutants in aqueous solutions was determined by UV–Vis spectrometry using UV–Vis equipment (Shimadzu UV-1700 PharmaSpec, Birmingham, United Kingdom). The spectrometric absorbance was monitored at the maximum wavelengths of 496.8 nm for CR dye and 282.5 nm for 2,4-D herbicide, respectively. The removal efficiency was assessed as given by:

(1) $$Y_{i}=\left(\frac{C_{0}-C_{t}}{C_{0}}\right)\times 100$$

where *C_0_* is the initial concentration of the pollutant (set to 50 mg/L), *C_t_*—concentration of the pollutant at the end of the test (at given time *t* = 120 min), and *i—*pollutant index (*i* = 1,2). Note that, designated as *Y_1_* and *Y_2_* were the removal efficiency of CR dye and 2,4-D pesticide, respectively.

In addition, the optimal material (which resulted from screening assay) was tested in a commercial photoreactor with larger volumetric capacity (*V_R_* = 600 mL). This commercial equipment represents an advanced UV-reactor of type Peschl Ultraviolet equipped with a TQ150 power box and a mercury lamp (immersive lighting of 150 W) that can be inserted and centered in the volume of the feed solution. In these experiments, the photocatalyst sample (0.1 g) was added to 600 mL of aqueous solution containing the pollutant with an initial concentration of 50 mg/L. Subsequently, the suspension was magnetically stirred (500 rotation/min) in the dark for 30 min to reach the adsorption–desorption equilibrium. Afterwards, the solution was exposed up to 120 min to UV-light irradiation. It should be noted that the lamp was surrounded by a circulating water jacket to cool down the reaction solution. The aliquots were extracted at different time intervals to evaluate analytically the change in the pollutant concentration during the photocatalytic reaction (kinetic decay). Finally, the UV–Vis spectrophotometer was employed to evaluate the absorbance in the range 200–800 nm, by registering UV–Vis spectra.

## 3. Results and Discussions

### 3.1. Design of Experiments and Multiple Regression Modeling

The synthesis of materials was planned according to the design of experiments in order to find out the relationship between factors affecting a process and the output of that process. In this sense, two variables were selected as key factors, namely: (1) *r*—the weight ratio of precursors, i.e., the ratio between the quantity of TTIP and the quantity of Pluronic F127; and (2) *t*—the sonication time (min) employed for materials synthesis. The actual and coded values of the design variables (key factors) used for materials synthesis are summarized in Table 1.

Considering these factors and their levels, a central composite design (of rotatable type) was adopted for the experimentation as given in Table 2. Note that, the design of experiments (DoE) and response surface methodology (RSM) are mathematical-statistical tools frequently used for the systematic investigation of systems and processes [18,19]. These tools enabled to study the synthesis process via simultaneous changing of the level factors, thereby employing a smaller number of experimental trials. According to DoE (Table 2), 11 runs of experimentation were carried out yielding nine types of materials (M1–M9). Note that, the runs no. 9, 10 and 11 were performed in the same conditions to test the reproducibility of the experiments, thereby producing the same type of the material (M9a, M9b and M9c). Each resulted material (M1–M9) according to the design was characterized in terms of surface area (*S_BET_*, m^2^/g) and pores volume (*V_P_*, cm^3^/g) (outputs) derived from N_2_-sorption isotherm, which values can be found in Table 2. The values of these characterization responses (*S_BET_* and *V_P_*) have been evaluated and it was found that the M4 sample exhibits the greatest *S_BET_* = 132.05 m^2^/g and *V_P_* = 0.331 cm^3^/g, following the trend of M1<M3<M2<M5<M7<M8<M9a<M6<M4 series, regarding the evolution of the BET specific surface area. As can be observed, not the same trend is followed by the samples, taking into account the total pore volume of the synthesized titania samples (M1<M3<M5<M7<M2<M9a<M8<M6<M4), the series being a little bit modified, but not to a large extent. Even so, the evaluated textural features place the M4 sample at the top of both series, expecting it to be a highly efficient material in adsorption and catalysis processes. Furthermore, each resulted material (M1–M9) was tested (screening assay) for the photodegradation of CR and 2,4-D pollutants using the small-capacity photoreactor (*V_R_* = 50 mL, under external UV-light irradiation). The responses derived from this application are summarized as well in Table 2 and were referred to as the removal efficiencies of CR dye (*Y_1_*, %) and 2,4-D herbicide (*Y_2_*, %).

On the basis of the experimental design and collected data (according to the Table 2), four multiple regression models were developed, being expressed in terms of coded variables as:

(2) $${\hat{S}}_{BET}=122.34+6.98x_{1}+2.01x_{2}+7.68x_{1}x_{2}-3.39x_{1}^{2}-3.17x_{2}^{2}$$

(3) $${\hat{V}}_{P}=0.29+0.016x_{1}+5.371\times 10^{-3}x_{2}+0.015x_{1}x_{2}$$

(4) $${\hat{Y}}_{1}=91.16-0.65x_{1}-1.20x_{2}+0.51x_{1}x_{2}+0.93x_{1}^{2}-2.86x_{2}^{2}$$

(5) $${\hat{Y}}_{2}=37.96-1.02x_{1}+1.03x_{2}+3.25x_{1}x_{2}+0.98x_{1}^{2}-0.86x_{2}^{2}$$

$$\mathrm{subject}\;\mathrm{to}:\;-\alpha \leq x_{i}\;\leq +\alpha ;\;\alpha =1.414;\;\forall i=\bar{1{,}_{}2}$$

where *x*_1_ and *x*_2_ are coded variables; ${\hat{S}}_{BET}$, ${\hat{V}}_{P}$, ${\hat{Y}}_{1}$ and ${\hat{Y}}_{2}$ denote predicted responses, respectively.

**Figure 1:**
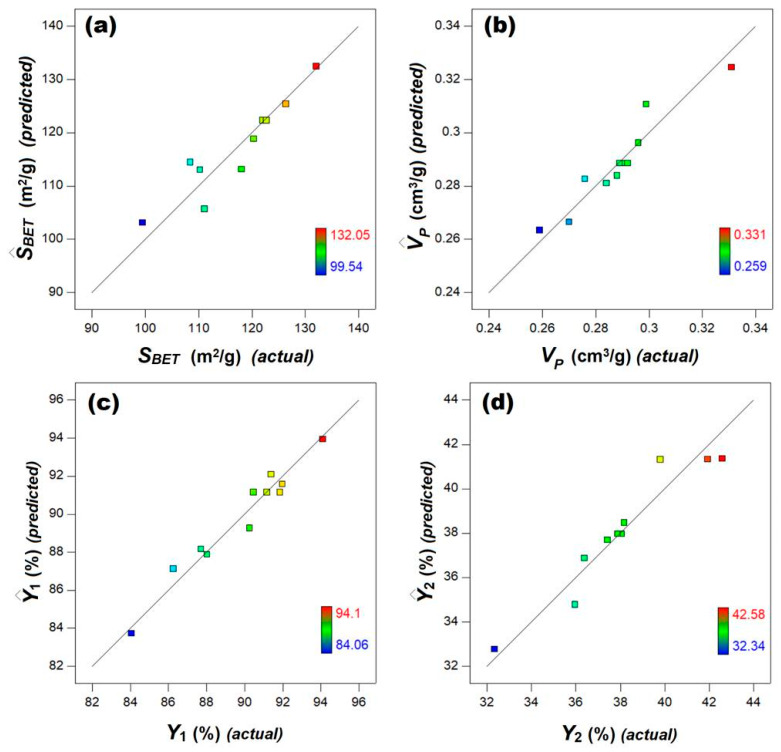
Parity plots showing the agreement between actual (observed) and predicted responses calculated according to fitted models; responses: (**a**) BET surface area; (**b**) volume of the pores; (**c**) removal efficiency of CR dye; (**d**) removal efficiency of 2,4 D pesticide.

Hence, the fitted models given in Equations (2)–(5) represent the second-order model and interaction equations implying the multiple regression coefficients. Each fitted model was statistically validated by the analysis of variance (ANOVA), which is detailed in the electronic supplemental information (ESI, Tables S1–S4). The agreements between predicted and observed responses are shown as parity plots in Figure 1a–d. As one can see, the data are in tolerable vicinity to bisectors, revealing that models are in reasonable agreement with the experimental observations. In summary, the parity plots (Figure 1) along with ANOVA (ESI, Tables S1 and S2) confirmed the statistical validation of the developed models. By using the mathematical substitution technique, the final empirical models in terms of actual factors can be expressed as follows:

(6) $${\hat{S}}_{BET}=108.84+1.036r-0.022t+0.051r\times t-0.135r^{2}-3.521\times 10^{-3}t^{2}$$

(7) $${\hat{V}}_{P}=0.306-2.874\times 10^{-3}r-8.209\times 10^{-4}t+1.00\times 10^{-4}r\times t$$

(8) $${\hat{Y}}_{1}=89.167-1.079r+0.308t+3.4\times 10^{-3}r\times t+0.037r^{2}-3.18\times 10^{-3}t^{2}$$

(9) $${\hat{Y}}_{2}=51.384-2.284r-0.067t+0.021r\times t+0.039r^{2}-9.602\times 10^{-4}t^{2}$$

subject to: 3 ≤ *r* ≤ 17; 18 ≤ *t* ≤ 102 (min)

where, *r* and *t* denote the actual factors of experimentation, i.e., *r*—weight ratio of the precursors (TTIP/surfactant), and *t*—sonication time (min).

**Figure 2:**
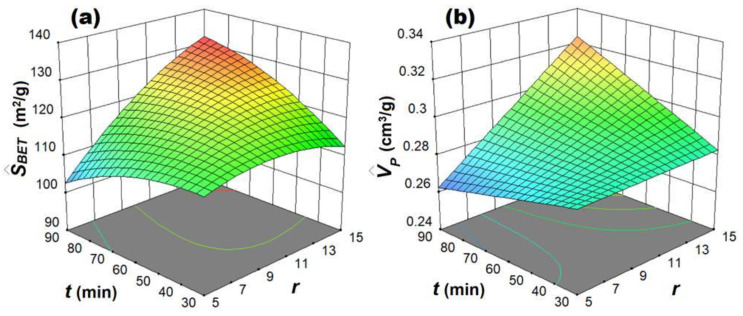
Surface plots of the responses derived from materials characterization by N_2_/BET adsorption isotherms: (**a**) BET surface area (*S*_BET_) depending on the factors (*t* and *r*); (**b**) volume of pores (*V*_P_) depending on the factors (*t* and *r*).

**Figure 3:**
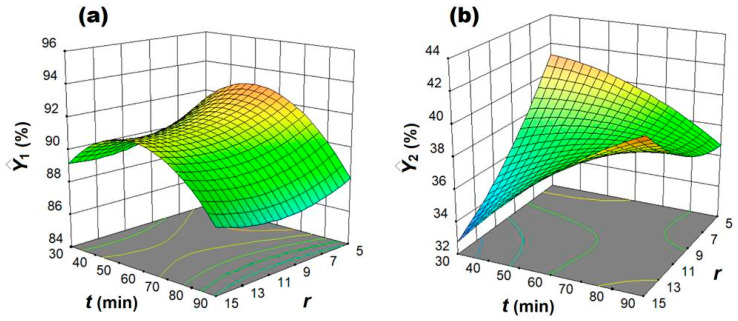
Surface plots of the responses derived from materials application in photodegradation: (**a**) removal efficiency of CR (*Y_1_*) depending on the factors (*t* and *r*); (**b**) removal efficiency of 2,4-D (*Y_2_*) depending on the factors (*t* and *r*).

Assuming empirical models given in Equations (6)–(9), we performed the computer-aided simulations to represent the material responses as functions of input variables (factors). Thus, Figure 2 and Figure 3 highlight the coupling effects of factors (*r* and *t*) on the responses of interest (${\hat{S}}_{BET}$, ${\hat{V}}_{P}$, ${\hat{Y}}_{1}$ and ${\hat{Y}}_{2}$). The combined effect of factors (*r* and *t*) on the characterization responses (${\hat{S}}_{BET}$ and ${\hat{V}}_{P}$) is depicted in Figure 2.

According to Figure 2, the increment of both factors (*r* and *t*) results in the increasing of the morphological responses. Hence, both 3D surfaces (given in Figure 2a,b) show similar trends. However, owing to quadratic terms in the nonlinear model, the response surface associated with the BET area (Figure 2a) implies a curvature effect. This effect does not appear for the response surface associated with the volume of pores (Figure 2b). Nevertheless, the interaction effect of factors persists in both cases. For instance, the increasing of the sonication time (*t*) at small ratio (*r*) conducts to slow decrease of the responses (${\hat{S}}_{BET}$ and ${\hat{V}}_{P}$). In turn, at a greater ratio (*r*), the increment of the sonication time (*t*) improves both morphological responses (Figure 2a,b). On the other hand, at low levels of the sonication time, the increasing of the ratio factor (*r*) does not affect considerably the values of responses. By contrast, at high levels of the sonication time, the increment of the ratio factor (*r*) conducts to significant increasing of responses values (Figure 2a,b). 

Figure 3 shows the influence of factors (*r* and *t*) on the removal efficiencies (${\hat{Y}}_{1}$ and ${\hat{Y}}_{2}$) that derived from the application of produced materials in photodegradation. As detailed, the response surfaces describe the removal efficiency of CR (${\hat{Y}}_{1}$, Figure 3a) and 2,4-D (${\hat{Y}}_{2}$, Figure 3b), both representing saddle-type surfaces. As one can see from Figure 3a, the increment of the sonication time conducts to the increasing, and then, to the decreasing of the response ${\hat{Y}}_{1}$ (i.e., curvature effect of *t*). Herein, the effect of the ratio factor (*r*) is also nonlinear, being less influencing than the sonication time.

The inspection of Figure 3b indicates a strong interaction effect between factors (*r* and *t*) for the case of 2,4-D degradation. Hence, as the sonication time is increased, at low ratio factor (*r*), the response ${\hat{Y}}_{2}$ is improved. At high ratio factor (*r*), the greater the sonication time, the less the response ${\hat{Y}}_{2}$ is. In addition, the increment of the ratio factor (*r*) at greater sonication time leads to the increasing of the response ${\hat{Y}}_{2}$. By contrast, at low sonication time, the increasing of the ratio factor (*r*) conducts to diminishing of the response (Figure 3b). 

For a saddle-type surface, there is an inflexion point (saddle point) between the relative minimum and maximum [P1]. For instance, the displacement from the saddle point along two opposite directions can improve or impair the response. Commonly, the visual inspection of the response surface can suggest the optimum region. According to Figure 3a, the optimal sonication time (for CR photodegradation) is pinpointed in the region of 50–70 min. In turn, the optimal sonication time (for 2,4-D photodegradation) can be extended to a larger region of 30–90 min (Figure 3b). The optimal precursors ratio (for CR photodegradation, see Figure 3a) can be distinguished at low levels (*r* ≤ 5). For the case of 2,4-D photodegradation (Figure 3b), the optimal ratio factor can be noticed at both low levels (*r* ≤ 5) and high levels (*r* ≥ 13), such a situation being typical for the saddle-type surfaces [20].

### 3.2. Multi-Objective Optimization

The objective of the design of experiments employed in this study was to figure out optimum conditions for the preparation of the material with improved photocatalytic performance. To select the optimal material from the produced set, we employed the desirability function approach (DFA) [18,21]. This method (DFA) is typically exploited to solve the multi-objective optimization problems, where the optimum should be decided based on several criteria (viewpoints). In this study, the optimization criteria of interest implied the enhanced photocatalytic performance and the proper morphology of the developed material. Consequently, we considered three responses for the multi-objective optimization, namely: (1) removal efficiency of CR dye, (2) removal efficiency of 2,4-D herbicide, and (3) BET surface area of the material. These responses were subjected to maximization. Thus, the optimal photocatalytic material should satisfy all these criteria combined into the best synergetic effect.

According to DFA method, the individual desirability functions (*d_i_*) must be calculated, firstly, by converting the actual values of responses (see Table 2) into the normalized values {0,1}. For responses subjected to maximization, the individual desirability functions can be ascertained by the conversion scheme of type LTB (the larger the best) that can be expressed as follows [18,21]:

(10) $$d_{i}=\left\{ \begin{array}{ll} 0, & \mathrm{if}\;\;\;Y_{i}\leq Y_{i}^{\alpha }\\ \left(\frac{Y_{i}-Y_{i}^{\alpha }}{Y_{i}^{\beta }-Y_{i}^{\alpha }}\right), & \mathrm{if}\;\;\;Y_{i}^{\alpha }\leq Y_{i}\leq Y_{i}^{\beta }\\ 1, & \mathrm{if}\;\;\;Y_{i}\geq Y_{i}^{\beta } \end{array}\right.$$

where *Y_i_* is the actual value of the response with index *i*; *Y_i_^α^* is the lower-bound limit and *Y_i_^β^* is the upper-bound limit of the response; *d_i_*—individual desirability of the response.

To assess the global desirability, the individual desirability function can be powered by a weight coefficient (importance) attributed to every single response. The value of the weight coefficient was equal to *w* = 1 (for less important response) and *w* = 2 (for more important response). In our case, the responses with more importance (*w*_1_ = 2 and *w*_2_ = 2) were the removal efficiencies of CR dye (*Y_1_*) and 2,4-D herbicide (*Y_2_*). In turn, the response with less importance (*w*_3_ = 1) was attributed to the BET surface area (*S_BET_*). Thus, more emphasis is placed on photodegradation performances rather than on the morphological characteristic of the material. Ultimately, the individual desirability functions (*d_i_*) were augmented into a composite function termed as the global desirability (*D*). This was calculated as the weighted geometric mean according to the next equation [18,21]:

(11) $$D={\left(\prod_{i=1}^{n}d_{i}{}^{w_{i}}\right)}^{\frac{1}{\sum w_{i}}}$$

For our case with three responses (*n* = 3) powered at different importance (i.e., *w*_1_ = 2, *w*_2_ = 2, and *w*_3_ = 1), Equation (11) can be written as:

(12) $$D=\sqrt[{5}]{d_{{}_{1}}^{2}\times d_{{}_{2}}^{2}\times d_{3}}$$

**Figure 4:**
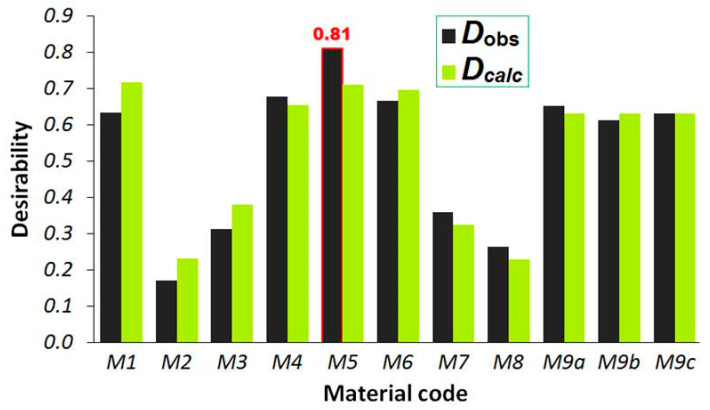
Global desirability values attributed to each material sample (observed values vs. calculated values).

Note that, the global desirability was calculated by taking into account the observed values of responses versus their predicted values. Thus, we calculated two global desirability indicators: (1) *D*_obs_—assuming actual responses, and (2) *D*_calc_—assuming predicted responses. Figure 4 compares the produced catalytic materials (M1–M9) in terms of global desirability. A reasonable agreement was noticed between the observed (*D*_obs_) and predicted (*D*_calc_) values of the global desirability (Figure 4).

As one can see from Figure 4, the optimum material for the photocatalytic application was M5 (produced at *r* = 3 and *t* = 60 min), which yielded the highest observable value of the global desirability (*D_obs_* = 0.81). Likewise, the material M5 showed the greatest removal efficiencies of CR (*Y_1_* = 94.10%) and 2,4-D (*Y_2_* = 42.58%) in the reaction system of *V_R_* = 50 mL capacity. Comparing to the sample M4, showing the best textural features, the optimal sample M5 revealed a smaller surface area, but a better photocatalytic activity, being in good agreement with their structural, textural, and optical properties.

Therefore, this optimum material M5 was retained for supplemental characterizations and additional studies (photodegradation kinetics).

### 3.3. Characteristics of Optimum Material

**Figure 5:**
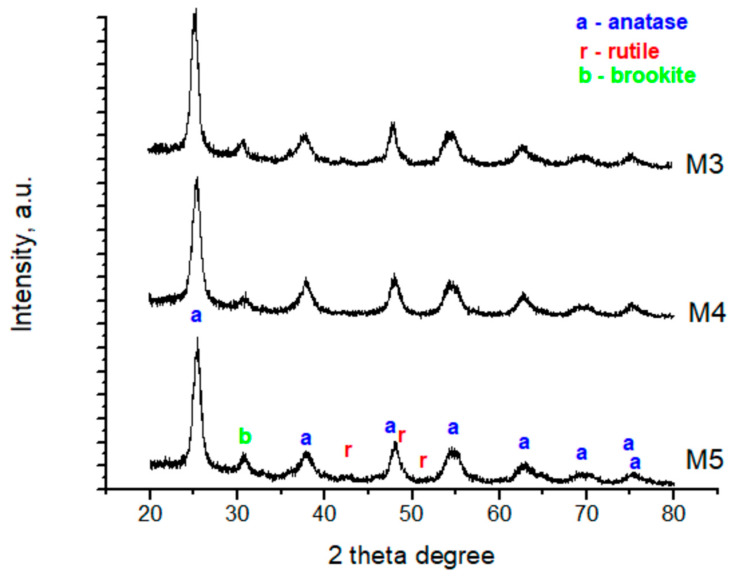
XRD diffractograms for M3, M4, and M5 TiO_2_ samples (the identification of rutile, anatase, and brookite phases according JCPDS card no. 01-073-1765, JCPDS card no. 01-089-4203, and JCPDS card no. 29-1360 respectively).

The optimal photocatalyst was characterized in terms of texture, structure, surface morphology, chemistry and the obtained results are detailed in Electronic Supporting Information (ESI). According to ESI, Figure S1, the hysteresis loop of the M5 sample is associated with H2 type (IUPAC classification) [22] and has a sharp pore emptying at *P/P_0_* = 0.7, meaning that the desorption of nitrogen occurs via cavitation mechanism. This indicates that the pores are of ink-bottle shape which gives the type H2 hysteresis due to pore percolation effect. Compared to the M4 sample, which exhibits a decreased slope of the desorption branch meaning a switched desorption mechanism to pore blocking, the M5 sample titania structure seems to contain pores with a narrower neck [23]. The XRD pattern (Figure 5) proves the formation of the crystalline anatase phase as the main phase [12,17], where the interplanar distance is of *d_101_* = 0.35 nm and the unit cell parameter is of *a_0_* = 0.40 nm (Table 3).

The crystallite size have been calculated as usual with the Scherrer formula (D_Scherrer_), but due to the XRD peak broadening that occurs due to the lattice strain (ξ), the Williamson–Hall equation have been considered to be more suited for crystallite size determination (D_W.H._) (Table 3). Analyzing the calculated values for the crystallite size by both equations, one can be observed that the M5 and M4 samples show not so different size values compared to the M3 sample. In addition, the strain value (ξ) is negative which may be due to lattice shrinkage in the case of the M4 and M5 samples.

**Figure 6:**
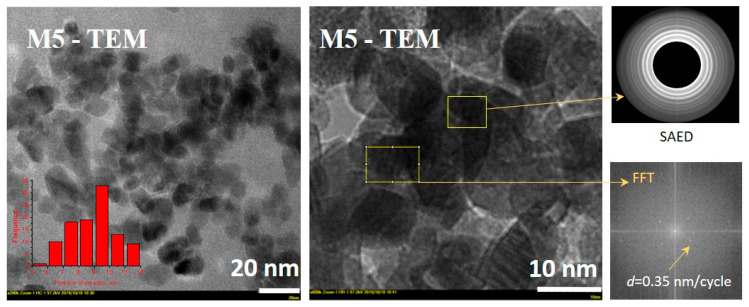
TEM images, particle size distribution plot (in red), selected area electron diffraction (SAED), and FFT for the synthesized TiO_2_ nanoparticles—M5 sample.

The powder morphology has been investigated by SEM imaging (ESI, Figure S2) showing more compact agglomerates in the case of the M5 sample, while the M4 titania sample exhibits fine dispersed powder. The mean crystallite size of the sample M5 was found to be of *D_TEM_* = 8.89 nm (the particle size distribution (in red) Figure 6), whose value is more or less the same as that calculated by Scherrer and Williamson–Hall equations using XRD diffraction patterns. As well, a very nice diffraction picture (TEM-derived SAED, Figure 6) has been acquired, indicating that the M5 sample have a great diffraction at low angles, indicating the titania pore structure is ordered. As it is very important to be known for the photocatalytic applications, the band gap energy of titania M5 sample has been calculated applying Tauc theory to the registered UVDR spectrum. Thus, from the UVDR-derived Tauc indirect plots, the band gap energy for the M5 titania sample has been found to be *E_g_* = 3.21 eV, compare to *E_g_* = 3.22 eV of the M4 sample (ESI, Figure S3). This band gap energy seems to be a suitable value for a semiconductor material capable to harvest the UV-light. Furthermore, the surface chemistry has been investigated by registering the FTIR spectrum of the M5 titania sample (ESI, Figure S4), which is a characteristic one, indicating a clear stretching vibration of the hydroxyl groups O-H on the titania nanoparticles (at 3500 cm^−1^), bending modes of water Ti-OH (at 1631 cm^−1^), and Ti-O modes (400–900 cm^−1^). No evident differences between M4 and M5 titania samples could be observed, meaning the variable factors considered in this research do not greatly affect the surface chemistry of the synthesized material.

### 3.4. Kinetics of the Photocatalytic Process

The heterogeneous photocatalytic degradation of water-soluble organic pollutants in the presence of inorganic oxidic catalysts can be expressed by the pseudo first-order kinetics. To reveal the kinetic of the photocatalytic process, a commercial photoreactor (Peschl Ultraviolet/TQ150, Mainz, Germany) with larger volumetric capacity (*V_R_* = 600 mL) has been employed. In a typical experiment, the optimum photocatalyst sample M5 (0.1 g TiO_2_) was added to 600 mL aqueous solution containing 50 mg/L of pollutant (initial concentration). The resulted suspension was magnetically stirred in the dark (for 30 min at 500 rpm) to reach the equilibrium. Then, the solution was irradiated up to 2 h under UV-light. It should be mentioned that the UV-lamp (surrounded by a circulating water jacket) was immersed in the center of the reaction solution. Samples were extracted periodically to monitor the pollutant concentration over the course of the photodegradation reaction. The UV–Vis absorption spectra for the extracted samples were recorded in the range of 200–700 nm (wavelength).

**Figure 7:**
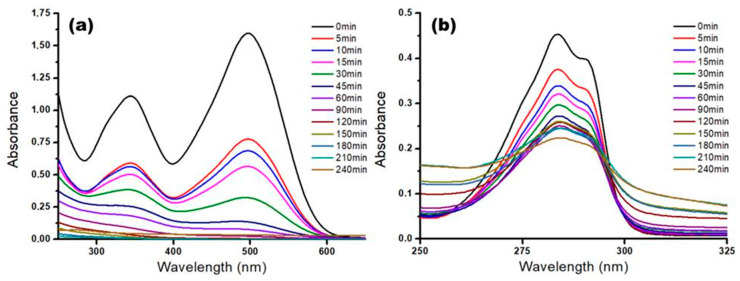
UV–Vis spectra profiles at various irradiation times accounting for the photodegradation reactions in the presence of the catalyst sample M5 (dosage = 0.167 g/L); (**a**) photodegradation of CR dye (50 mg L^−1^); (**b**) photodegradation of 2,4-D herbicide (50 mg L^−1^).

Figure 7 shows the evolution of the UV–Vis absorbance spectra for CR (Figure 7a) and 2,4-D (Figure 7b) solutions after irradiation by UV-light in the presence of the M5 photocatalyst. As one can see, in both cases, the intensity of the absorption peaks was diminished with the increment of the irradiation time (Figure 7).

**Figure 8:**
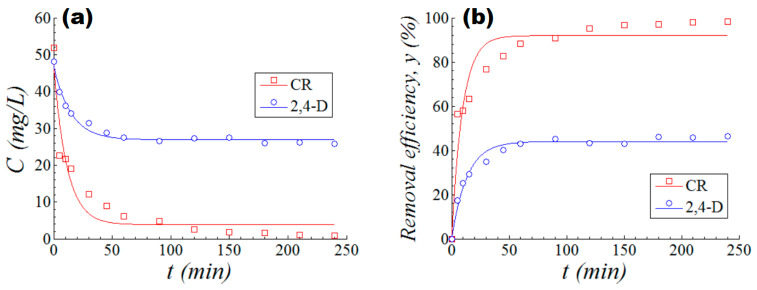
Photodegradation of organic pollutants in aqueous solutions in the presence of the M5 catalyst sample; (**a**) concentration decay of CR and 2,4-D pollutants against reaction time; (**b**) removal efficiency evolution on the course of the photodegradation; conditions: initial pollutant concentration 50.0 ± 0.5 mg/L, dosage of catalyst 0.167 g/L, *T* = 20 ± 2 °C, pH 6.8 ± 0.2, *V_R_* = 600 mL; (solid lines denote predictions given by pseudo first-order kinetic models).

Assuming the calibration curves, the decay of the pollutants’ concentrations and the evolution of the removal efficiencies have been determined. The resulted kinetic data were interpolated by pseudo first-order models using nonlinear regression techniques. In this regard, the time evolutions of the pollutants’ concentrations, and respective removal efficiencies, were fitted to the pseudo first-order kinetic models with the residual (stable) component [24,25], as given by:

(13) $$C\left(t\right)=\left(C_{0}-C_{e}\right)\times e^{-kt}+C_{e}$$

(14) $$Y(t)=Y_{e}\times \left(1-e^{-\gamma t}\right)$$

where *C_0_* denotes the initial pollutant concentration (50 mg/L), *C_e_*—residual pollutant concentration (mg/L), *k*—pseudo first-order reaction rate constant (min^−1^), *t*—irradiation time (min); *γ*—pseudo first-order removal rate constant (min^−1^); *Y_e_*—removal efficiency (%) emerged due to the residual pollutant concentration (*C_e_*). Note that, the residual (stable) component (*C_e_*) is a portion of the initial pollutant concentration, which is extremely persistent [25]. According to Figure 8a,b (as well to ESI, Figure S5), the experimental data were in reasonable agreement with the predictions given by pseudo first-order kinetic models.

The calculated kinetic parameters are summarized in Table 4. As detailed, the values of pseudo first-order rate constants (*k* and *γ*) were greater for the system CR + M5, if compared to the system 2,4-D+M5. Thus, the photodegradation of CR dye (*k_1_* = 8.86 × 10^−2^ min^−1^) was about 1.3-fold faster than the photodegradation of 2,4-D pesticide (*k_2_* = 6.84 × 10^−2^ min^−1^), keeping the same trend of the photodegradation with the external UV-lamp, even if that was less powerful one (6 vs. 150 W). Note that, the ratios between rate constants were found to be quite similar, i.e., (*k*_1_/*k*_2_) ≈ (*γ*_1_/*γ*_2_) (see Table 4). The final removal efficiency (after *t* = 120 min photodegradation) was equal to 98.40% and 46.30%, for CR and 2,4-D, respectively. These values (98.40% and 46.40%) were the greatest ones determined for this study. Hence, the degradation of pollutants in the commercial photoreactor (*V_R_* = 600 mL) equipped with the internal UV-lamp was more intense (by 4% in both cases) than the degradation of pollutants in the smaller-sized photoreactor (*V_R_* = 50 mL) equipped with the external UV-lamp.

## 4. Conclusions

Herein, the optimization of the ultrasound-assisted synthesis process of mesoporous TiO_2_ has been successfully implemented. The empirical model and optimization have been applied to the synthesis process using the response surface methodology (RSM). Thus, the effects of two experimental factors were assessed in the synthesis process, as the weight ratio of precursors (*r*) and the sonication time (*t*), being employed in the design of experiments (DoE) to develop data-driven models and to optimize conditions of the synthesis process.

Regarding the photocatalytic activity of the prepared TiO_2_, the degradation of Congo red dye and 2,4-D herbicide under UV–Vis irradiation has been investigated. The optimal synthetic conditions for the efficient pollutants’ photodegradation were found when 3 grams of Pluronic F127 and 60 min of the sonication time have been considered. Thus, the optimal produced TiO_2_ material (M5 sample) under these optimal conditions yielded the maximal removal efficiencies of 98.4% (CR removal) and 46.3% (2,4-D removal), when an immersive UV-lamp has been used. The photodegradation kinetics disclosed the pseudo first-order rate constants equal to *k_1_* = 8.86 × 10^−2^ (min^−1^) and *k_2_* =6.84 × 10^−2^ (min^−1^) associated to the systems (TiO_2_ + CR) and (TiO_2_ + 2,4-D), respectively. In conclusion, the synergism between the crystal size, specific surface area, defect population and porosity, factors working together that influence the rate of recombination of the holes and the electrons in titania photocatalyst, was found to be effective in the photodegradation process.

## Figures and Tables

**Table 1:** Design variables and their coded and actual values used for materials synthesis.

Design Variables (Factors)	Coded Variables	Actual Values of Coded Levels				
		−α	−1	0	+1	+α
Ratio of precursors’ weights (TTIP/Surfactant), *r*	*x*_1_	3	5	10	15	17
Sonication time, *t* (min)	*x*_2_	18	30	60	90	102

*Note*: α = 1.414 (value of axial point for rotatable CCD in case of two factors).

**Table 2:** Central composite design (rotatable type) used for the experimentation; (materials’ synthesis, characterization and application in photodegradation); α = 1.414.

Run	Design Variables				Resulted Material (Code)	Responses (Material Characterization) ^1^ & (Application) ^2^			
	Ratio of Precursors		Sonication Time (min)						
	*x*_1_	*r*	*x*_2_	*t*		*S*_BET_ (m^2^/g) ^1^	*V*_P_ (cm^3^/g) ^1^	*Y*_1_ (%) ^2^	*Y*_2_ (%) ^2^
1	−1	5	−1	30	M1	108.46	0.276	91.98	39.80
2	+1	15	−1	30	M2	110.26	0.288	90.26	32.34
3	−1	5	+1	90	M3	99.54	0.259	87.71	36.39
4	+1	15	+1	90	M4	132.05	0.331	88.03	41.93
5	−α	3	0	60	M5	111.15	0.270	94.10	42.58
6	+α	17	0	60	M6	126.36	0.299	91.39	38.17
7	0	10	−α	18	M7	118.06	0.284	86.25	35.96
8	0	10	+α	102	M8	120.33	0.296	84.06	37.43
9	0	10	0	60	M9a	122.32	0.291	91.85	37.96
10	0	10	0	60	M9b	121.97	0.292	90.46	38.05
11	0	10	0	60	M9c	122.71	0.289	91.16	37.88

^1^—responses derived from characterization of materials: adsorption isotherm parameters (N_2_/BET). ^2^—responses derived from application of materials in photodegradation: removal efficiencies determined in the small equipment (*V_R_* = 50 mL) with external UV–Vis lamp.

**Table 3:** Structural parameters calculated using XRD patterns of the selected titania samples.

Sample	D_Scherrer_ (nm)	a (nm)	d (nm)	D_W.H._ (nm)	ξ
M3	10.375	0.351302	0.405649	18.561	0.0372
M4	9.156	0.351191	0.405520	8.803	-0.0002
M5	9.701	0.351055	0.405363	9.064	−6E−05

D_Scherrer_ (nm): crystallite size calculated by using Scherrer formula; a (nm): elemental cell parameter; d (nm): interplanar distance; D_W.H._ (nm): crystallite size calculated by using Williamson–Hall approximation; ξ: strain (slope from Williamson–Hall plot).

**Table 4:** Kinetic parameters for Congo-Red dye and 2,4-D photodegradation in the presence of the catalyst sample M5; catalyst dosage = 0.167 g/L; *V_R_* = 600 mL; *T* = 20 ± 2 °C; pH 6.0 ± 0.5.

No. (*i*)	Photocatalytic System	Pseudo first-order Reaction Rate Constant, *k_i_* (min^−1^)	Pseudo first-order Removal Rate Constant, *γ_i_* (min^−1^)	Final Removal Efficiency (at *t* = 120 min) Y* (%)
1	M5 + CR dye	8.86 × 10^−2^	10.59 × 10^−2^	98.40%
2	M5 + 2,4-D	6.84 × 10^−2^	7.51 × 10^−2^	46.30%

*Note*: *k*_1_/*k*_2_ = 1.3 vs. *γ*_1_/*γ*_2_ = 1.4. * optimum value.

## Supplementary Materials

The following are available online at https://www.mdpi.com/2079-4991/10/5/998/s1, Table S1: ANOVA test for the fitted model; Table S2: ANOVA test for the fitted model; Table S3: ANOVA test for the fitted model; Table S4: ANOVA test for the fitted model; Figure S1: Nitrogen adsorption–desorption isotherms, and corresponding BJH pore size distributions (calculated from adsorption and desorption branches, respectively); Figure S2: SEM images for the synthesized TiO_2_ nanoparticles—M4 and M5 samples; Figure S3: UVDR-derived Tauc indirect plots for the synthesized TiO_2_ nanoparticles—M4 and M5 samples, and determined band gap energies; Figure S4: FTIR spectra of synthesized TiO_2_ nanoparticles—M4 and M5 samples.
