# Supplementary material for: Development of Porous Titania Structure with Improved Photocatalytic Activity: Response Surface Modeling and Multi-Objective Optimization
Source: Nanomaterials (Basel). 2020 May 23;10(5):998. doi: 10.3390/nano10050998 (PMC7279558; doi:10.3390/nano10050998)
Supplement: Supplementary file 1 [file nanomaterials-10-00998-s001.zip › nanomaterials-804134-supplementary.docx]

***Electronic Supporting Information (ESI)***

**Development of porous titania structure with improved photocatalytic activity: Response surface modeling and multi-objective optimization**

Elvira Mahu, Maria Ignat *, Corneliu Cojocaru *, Petrisor Samoila, Cristina Coromelci, Iuliean Asaftei, Valeria Harabagiu

*submitted to:*

**NANOMATERIALS**

**Analysis of Variance (ANOVA) to test the significance of fitted models**

**Table S1**. ANOVA test for the fitted model.

| Source | *DF* (a) | *SS* (b) | *MS* (c) | *F-value* (d) | *P-value* (e) | *R2* (f) | *Radj 2* (g) |
| --- | --- | --- | --- | --- | --- | --- | --- |
| Model | 5 | 751.72 | 150.34 | 6.58 | 0.0296 | 0.868 | 0.736 |
| Residual | 5 | 114.28 | 22.86 |  |  |  |  |
| Total | 10 | 866.00 |  |  |  |  |  |

**Table S2**. ANOVA test for the fitted model .

| Source | *DF* | *SS* | *MS* | *F-value* | *P-value* | *R2* | *Radj 2* |
| --- | --- | --- | --- | --- | --- | --- | --- |
| Model | 3 | 3.084×10-3 | 1.028×10-3 | 24.29 | 0.0004 | 0.912 | 0.875 |
| Residual | 7 | 2.962×10-4 | 4.232×10-5 |  |  |  |  |
| Total | 10 | 3.381×10-3 |  |  |  |  |  |

**Table S3**. ANOVA test for the fitted model .

| Source | *DF* (a) | *SS* (b) | *MS* (c) | *F-value* (d) | *P-value* (e) | *R2* (f) | *Radj 2* (g) |
| --- | --- | --- | --- | --- | --- | --- | --- |
| Model | 5 | 81.75 | 16.35 | 21.82 | 0.0021 | 0.956 | 0.912 |
| Residual | 5 | 3.75 | 0.75 |  |  |  |  |
| Total | 10 | 85.49 |  |  |  |  |  |

**Table S4**. ANOVA test for the fitted model .

| Source | *DF* (a) | *SS* (b) | *MS* (c) | *F-value* (d) | *P-value* (e) | *R2* (f) | *Radj 2* (g) |
| --- | --- | --- | --- | --- | --- | --- | --- |
| Model | 5 | 72.66 | 14.53 | 11.84 | 0.0084 | 0.922 | 0.844 |
| Residual | 5 | 6.14 | 1.23 |  |  |  |  |
| Total | 10 | 78.80 |  |  |  |  |  |

(a) degree of freedom; (b) sum of squares; (c) mean square; (d) ratio between mean squares;

(e) probability of randomness; (f) coefficient of determination; (g) adjusted coefficient of determination;

As probability values (*P-value)* are less than 0.05 and the values of determination coefficient (*R*2) tend to unity (*R*2🡪1), the developed models are statistically significant and can be used to navigate the design space.

**
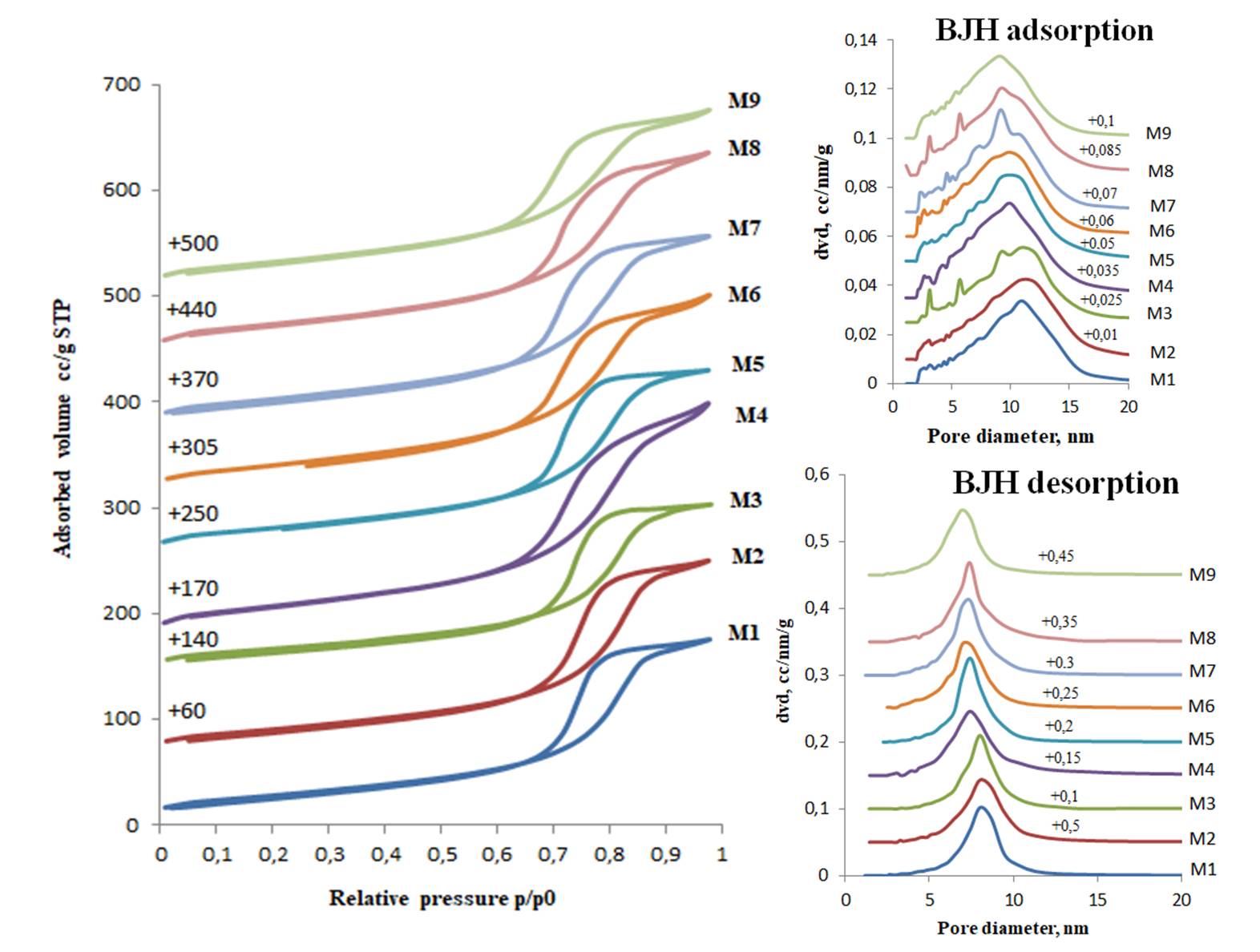
**

**Fig.S1**. Nitrogen adsorption-desorption isotherms, and corresponding BJH pore size distributions (calculated from adsorption and desorption branches, respectively).


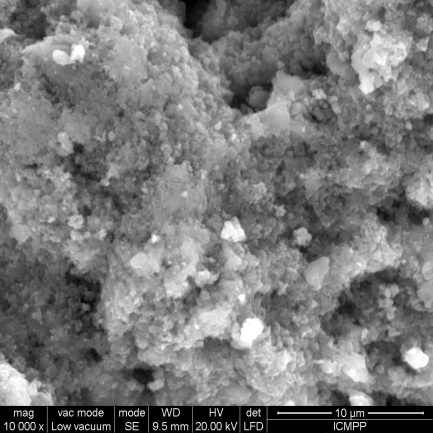

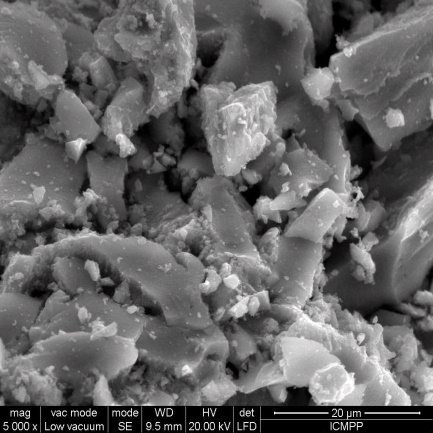

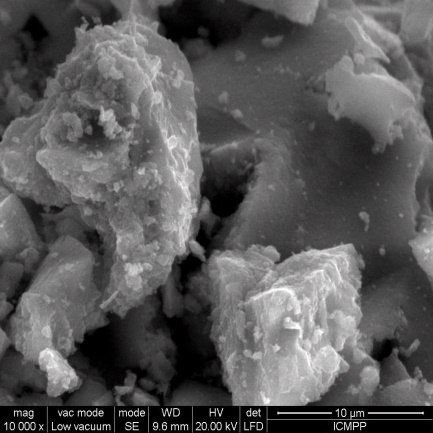

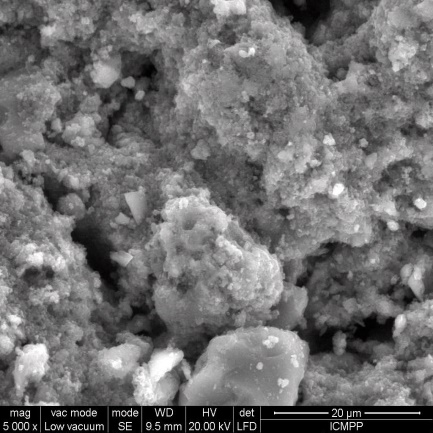


**20 µm**

**10 µm**

**20 µm**

**10 µm**

**M4 - SEM**

**M4 - SEM**

**M5 - SEM**

**M5 - SEM**

**Fig.S2**. SEM images for the synthesized TiO2 nanoparticles - M4 and M5 samples.


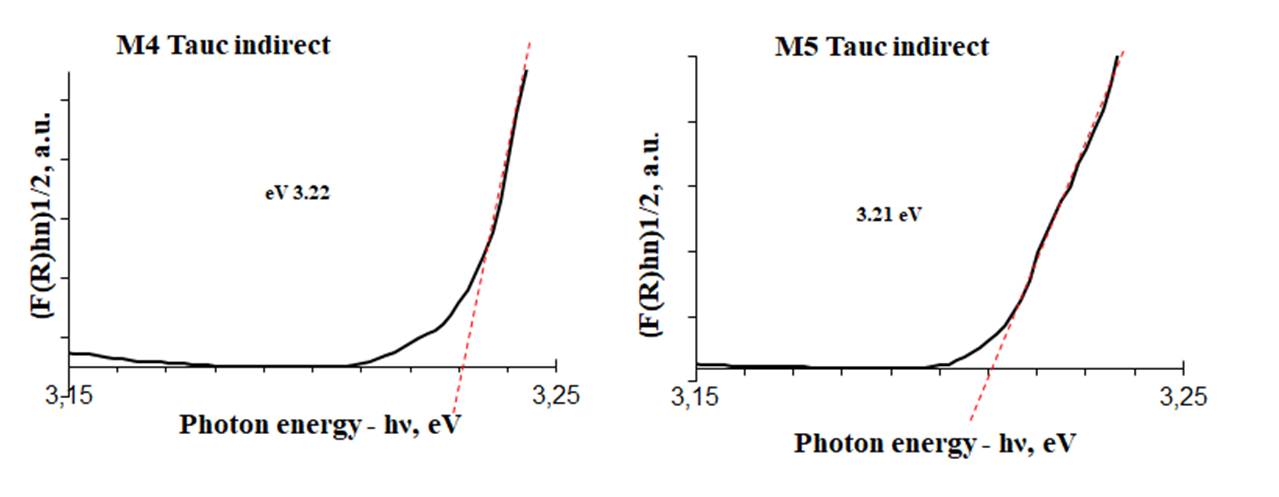
 **Fig.S3**. UVDR-derived Tauc indirect plots for the synthesized TiO2 nanoparticles - M4 and M5 samples, and determined band gap energies.

**Fig.S4**. FTIR spectra of synthesized TiO2 nanoparticles - M4 and M5 samples.

**Fig.S5** Plots of ln(C/Co) as a function of time for the reduction of CR dye and 2,4D pesticide catalyzed by M5 TiO2 sample.
